# Oral health in migrant population of Germany: a systematic review and meta-analysis

**DOI:** 10.1186/s12889-026-27927-8

**Published:** 2026-06-08

**Authors:** Deniz Kosan, Zhen Mao, Jamila Yassine, Esra Kosan

**Affiliations:** 1 Medical School Berlin (MSB), Berlin, Germany; 2 Department of Prosthodontics, Charité-Universitätsmedizin Berlin, Berlin, Germany; 3 Department of Periodontology, Oral Medicine and Oral Surgery, Charité-Universitätsmedizin Berlin, Aßmannshauser Str. 4-6, 14197, Berlin, Germany

**Keywords:** Oral Health, Migrant Health, Systematic Review, Health Equity, Oral Epidemiology

## Abstract

**Objective:**

Migrants living in Germany constitute a large and heterogeneous population that is exposed to socioeconomic disadvantages, language barriers and difficulties navigating the health system, all of which may negatively affect oral health and access to dental care. The aim of this study was to systematically review and synthesize available evidence on differences in oral health status and dental service utilization between migrant and non-migrant populations in Germany.

**Methods:**

A systematic review of peer-reviewed observational and clinical studies conducted in Germany was performed. Studies meeting predefined inclusion criteria were selected, focusing on oral health indicators such as dental caries prevalence (DMFT scores), periodontal health, and access to preventive dental care. Data on study characteristics, population demographics and outcomes were extracted using a standardized form. Where at least three sufficiently homogeneous studies were available, a meta-analysis of DMFT outcomes was conducted.

**Results:**

Seventeen studies were included in the qualitative synthesis and three in the meta-analysis. Across adult populations, migrants consistently showed worse oral health than non-migrants, with higher DMFT scores (SMD = 0.40; 95% CI: 0.31–0.49; *p* < 0.05) scores than non-migrants, driven primarily by a higher number of missing teeth (M) (SMD = 0.17; 95% CI: 0.08–0.26; *p* < 0.05), while differences in decayed (D) and filled (F) teeth were smaller and more heterogeneous. Children and adolescents with a migration background, particularly those living in socioeconomically disadvantaged areas, had higher caries experience and a lower probability of caries-free dentitions in permanent teeth. Evidence on caries in primary dentition (dmft) was limited and heterogeneous and therefore summarized qualitatively only. Across age groups, migrants had lower odds of attending regular preventive dental check-ups than non-migrants (OR approximately 0.64–0.67).

**Discussion:**

Despite statutory health insurance in Germany, migrants struggle with access to dental services. Structural and social determinants, including socioeconomic position, health literacy, language barriers, and perceived discrimination, likely contribute to these disparities.

**Conclusion:**

Targeted public health strategies, culturally and linguistically adapted information, and measures to improve accessibility of dental care are needed to reduce oral health inequalities between migrants and the native German population.

**Supplementary Information:**

The online version contains supplementary material available at 10.1186/s12889-026-27927-8.

## Introduction

Migration has become a defining demographic feature of Germany: in 2022 roughly one-quarter of the population had a history of immigration [1] and in 2023 nearly thirty percent were reported to have a migration background [2]. This population includes first-generation immigrants (born abroad), second-generation individuals born in Germany to at least one parent born abroad, and persons with refugee or asylum-seeker status. Migrants in Germany are highly heterogeneous in terms of country of origin, socioeconomic position, legal status and integration experience; factors that may influence oral health and dental-service use.

Oral health is a crucial component of overall well-being and quality of life. In Germany however, migrants often face a range of health disparities, exacerbated by social, cultural, and economic barriers. These barriers can hinder access to dental care and increase the risk of oral diseases [3].

In Germany, access to dental care is largely determined by statutory health insurance (SHI), which covers around 90% of the population. Migrants with legal residency permits, as well as individuals who acquire German nationality, are eligible for statutory health insurance under German law and therefore have the same entitlements to dental services as native German citizens [4]. Individuals who are granted asylum also transition to SHI coverage once their protection status is formally recognised, which provides access to standard dental care [5]. In contrast, asylum seekers during their initial application period receive healthcare under the Asylbewerberleistungsgesetz (Asylum Seekers’ Benefits Act), which limits dental care mainly to emergency treatment and the management of acute pain, with restricted access to preventive or restorative procedures [6]. Since 2022, refugees from Ukraine have been granted immediate access to SHI under the EU Temporary Protection Directive, regardless of their specific asylum status, aligning their dental care entitlements with those of other insured residents [7].

Previous studies have indicated that migrants in Germany tend to have poorer oral health and lower access to dental services compared to the native population. Contributing factors include language barriers, differing health beliefs, and socioeconomic status. For instance, a systematic review from 2022 highlighted that migrants and ethnic minority populations generally exhibit lower oral health literacy, which is associated with negative oral health behaviours and reduced dental service utilization [8].

Despite these findings, there is a limited number of systematic reviews that comprehensively examine the oral health status and the underlying determinants in this population group. A systematic review focusing on migrants from middle- and low-income countries to Europe reported a higher prevalence of dental caries and poorer periodontal health among migrants compared to non-migrant populations. The review also identified significant barriers to accessing dental care services, such as economic constraints and social disparities [9].

Oral health inequalities between migrant and non-migrant populations have been documented across Europe. However, evidence remains fragmented and highly context dependent. Germany represents a particularly important case for examining these disparities. As one of the largest host countries for migrants in Europe, Germany combines a heterogeneous migrant population with a universal healthcare system and comprehensive statutory dental coverage. Persistent differences in oral health outcomes and preventive dental service utilization in such a setting raise critical questions about structural, social, and cultural barriers that extend beyond formal insurance entitlements.

The aim of this study was therefore to systematically review and synthesize available evidence on differences in oral health status and dental service utilization between migrant and non-migrant populations in Germany. To achieve this, we conducted a systematic review with meta-analysis, in which the meta-analysis constitutes a quantitative component embedded within a broader narrative synthesis. While meta-analytic methods were used to quantify differences in recurrent outcomes such as the DMFT index and its components, the review also integrates contextual and qualitative findings to interpret observed disparities and situate the German evidence within an international perspective.

## Methods

### Protocol and registration

The systematic review was written according to the Preferred Reporting Items for Systematic Reviews and Meta-Analyses (PRISMA) guidelines [10]. The review protocol was established before the initiation of the research and registered in the International Prospective Register of Systematic Reviews (PROSPERO) database (CRD420251027726).

### Original review question and subsequent modification

The original question was: *How does the oral health status of the migrant population (P) compare to that of the German population (C) in terms of dental caries risk, periodontal disease prevalence and frequency of dental check-ups (I/O)?*

### Definitions

The European Commission defines an *Immigrant* as a person who establishes their usual residence in the territory of an EU Member State for a period of at least 12 months, after previously residing in another EU Member State or a third country. In contrast, the native population refers to individuals who were born in the country where they have their usual residence. Furthermore, the European Commission employs specific terminology to describe individuals based on their migration background and the birthplace of their parents:

- *Second-Generation Migrant:* A person who was born in and is residing in a country that at least one of their parents previously entered as a migrant.
- *Person with a Migratory Background:* This broad definition encompasses all individuals who have undertaken migration themselves and/or have parents who did so.

These definitions assist in distinguishing between various population groups within the European Union, particularly concerning migration statistics and policy development. We opted to include both immigrants and second-generation migrant under the term ‘migrant’, due to the assumption that the influence of a migrant parent might outweigh the socialisation in the birth country.

### Search strategy

A comprehensive literature search was conducted using multiple electronic databases: PubMed, Scopus and Web of Science, to identify relevant studies on oral health in the migrant population in Germany. The search was restricted to articles published from 1990 to the 31 st Febuary 2025. To ensure the inclusion of the most recent evidence, the literature search was updated on 3 January 2026 using the same databases, search terms, and inclusion criteria as the original search. Search terms and Medical Subject Headings (MeSH) can be found in Appendix Tab. 2.

### Inclusion criteria

The following inclusion criteria were applied.

1. Study design: Original research studies using observational designs (e.g., cross-sectional, cohort) that reported comparative data between migrants and native Germans.
2. Population: Studies including children and/or adults with a migration background living in Germany, reporting outcomes on oral health status or dental service utilisation.
3. Language: Articles published in English or German.
4. Location: Studies conducted exclusively in Germany.

### Exclusion criteria

The following exclusion criteria were applied:

1. Review articles, case reports, in vitro and animal studies.
2. Randomized controlled trials (RCTs) were generally excluded because their design and purpose did not align with the aim of this review. However, RCTs were eligible if they reported baseline (pre-intervention) oral health data separately for migrant and non-migrant participants. In such cases, only the baseline, pre-randomization data were extracted and used in the analysis, and no post-intervention outcomes were considered.
3. Studies published in other languages.
4. Studies with insufficient information about measurement method, subject number, follow-up period or unclear oral health outcomes.
5. When there were multiple publications on the same cohort of patients, only the manuscript reporting complete and required data was included.

### Study selection

The study selection process was carried out by two independent reviewers (EK, DK). Titles and abstracts of all identified studies were screened for relevance. Full-text articles of potentially eligible studies were retrieved and assessed for inclusion based on the predefined criteria. Any disagreements between the reviewers regarding study inclusion were resolved through discussion, and, if necessary, a third reviewer (ZM) was consulted.

### Data extraction

Data from the included studies were extracted independently by the two reviewers (EK, DK) using a standardized data extraction form. The extracted information included study characteristics (e.g., author, year of publication, study design), population demographics (e.g., age, gender, migrant status), methods, and key findings related to oral health outcomes. Particular attention was given to studies that provided comparative data between the migrant and German populations.

### Quality assessment

The methodological quality of the included studies was assessed using appropriate tools depending on the study design. Observational and cross-sectional studies were assessed using the Appraisal tool for Cross-Sectional Studies (AXIS) [11]. Since only baseline data were included and no intervention components were analyzed, the study by Lieske et al*.* [12] was assessed using the AXIS tool for cross-sectional studies rather than ROB-2. Tool. Discrepancies in quality assessment were resolved through consensus between the reviewers.

This systematic approach ensured the inclusion of high-quality, relevant studies, including those that provide comparative insights into oral health between the migrant and German populations, to offer a comprehensive understanding of disparities and influencing factors.

#### Certainty of evidence

The certainty of evidence for the quantitative outcomes included in this review was assessed using the Grading of Recommendations Assessment, Development and Evaluation (GRADE) approach. GRADE [13] provides a systematic and transparent framework for rating the quality of evidence across studies based on five key domains: risk of bias, inconsistency, indirectness, imprecision, and publication bias. Each outcome was evaluated and categorized into one of four certainty levels: high, moderate, low, or very low. Studies with qualitative or narrative designs, while excluded from the GRADE assessment due to methodological incommensurability, were included in the broader synthesis to provide contextual depth and support the interpretation of findings.

#### Data synthesis strategy

All data was analysed qualitatively and quantitatively, and a narrative synthesis of the included studies was performed. Meta-analysis was performed using R statistical software (R Foundation for Statistical Computing, Vienna, Austria) employing standard meta-analysis packages. for one recurrent outcome: the difference in DMFT index (Decayed, Missing, Filled Teeth) between German migrant and non-migrant groups. The outcomes during the follow-up period were pooled and analysed directly using standard mean differences (SMDs) and 95% confidence intervals in a random or fixed-effect model (Dersimonian-Laird test) depending on the heterogeneity. Heterogeneity between studies was assessed using a Q-test and the I^2^ value statistic. If I^2^ > 50%, heterogeneity was found and a random-effect model was used, otherwise fixed-effect was reported. A positive value stood for the higher outcomes was observed in migrant group, and vice versa. The inconsistent effects among each study were estimated by Forest plots. A value of I^2^ > 75% was regarded as high heterogeneity [14]. Sensitivity analysis were performed to investigate the possible variables causing heterogeneity. Statistical significance was defined as *p*-value < 0.05. Given that only three studies were included in the meta-analysis, formal assessment of publication bias (e.g., using funnel plots or Egger’s test) was not performed, as such methods are unreliable with small sample sizes. When RCTs were included based on baseline data, these baseline measurements were treated equivalently to observational cross-sectional data for the purposes of quantitative synthesis. No randomization-related effects, trial arms, or intervention outcomes were analysed.

## Results

### Study selection

**Fig. 1:**
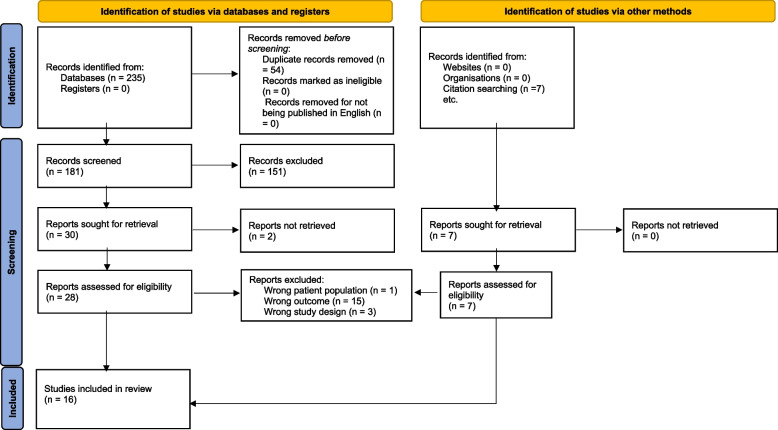
PRISMA 2020 flow diagram for new systematic reviews which included searches of databases, registers and other sources

The article selection process is summarized in Fig. 1. A total of 242 records were identified from the selected databases as well as through citation search. After the automatic removal of 54 duplicates, 188 articles remained for title and abstract screening. Of these, 37 studies were deemed eligible for full-text review. Two full-text articles could not be retrieved. Among the remaining 35 studies, one was excluded due to an irrelevant study population, 15 for reporting wrong outcomes, and three due to inappropriate study design (Table 1). Ultimately, 16 studies were included in the systematic review, and three of these were incorporated into the meta-analysis. One RCT (Lieske et al. [12]) was included exclusively for baseline, pre-intervention data because these data represent observational, pre-randomization characteristics and were directly comparable to the outcomes in the observational studies.

**Table 1:** Excluded studies and reasons for exclusion

Excluded Studies	Reason for Exclusion
Razum et al. 2016 [15]	Wrong outcome*
Ferrera et al. 2024 [16]	Wrong outcome
Kofahl et al. 2009 [17]	Wrong outcome
Sauter et al. 2021 [18]	Wrong outcome
Wenner et al. 2016 [19]	Wrong outcome
Führer et al. 2019 [20]	Wrong outcome
Brzoska et al. 2015 [21]	Wrong outcome
Walter et al. 2017 [22]	Wrong outcome
Holtfreter et al. 2012 [23]	Wrong outcome
Ciupitu et al. 2011 [24]	Wrong outcome
Bermejo et al. 2012 [25]	Wrong outcome
Wiegand et al. 2013 [26]	Wrong outcome
Kühnisch et al. 1998 [27]	Full text not found
Ramsak et al. 2023 [28]	Wrong outcome
Grieger et al. 2003 [29]	Wrong outcome
Klein et al. 2018 [30]	Wrong Study Design
Pistorius et al. 2003 [31]	Wrong Patient Population
Huber et al. 2012 [32]	Wrong Outcomes
Schäfer 1996 [33]	Full text not found
Spallek et al. 2010 [34]	Wrong Study Design
Wagner and Heinrich-Weltzien 2017 [35]	Wrong Study Design

^*^Wrong outcome refers to studies that did not report clinical oral health indicators (e.g. DMFT/DMFS, periodontal measures), dental service utilization or other predefined outcomes relevant to the review question

The inter-rater agreement, measured by the kappa coefficient, was 0.86 for the title/abstract screening and 0.82 for the full-text screening, indicating substantial to almost perfect agreement.

### Characteristics of the included studies

The included studies (Appendix, Tab. 1) consistently highlight substantial disparities in oral health status and dental care utilization between migrants and non-migrants in Germany. These inequalities are shaped by intersecting factors such as socioeconomic status, language proficiency, cultural perceptions, and health system navigation challenges. For instance, Aarabi et al. [36] found that elderly first-generation migrants had significantly higher DMFT scores and fewer filled teeth than their non-migrant counterparts, along with reported difficulties in accessing care due to cost and language barriers. Bissar et al. (2007a, 2007b) [37, 38] focused on schoolchildren in socioeconomically disadvantaged areas and reported significantly higher levels of untreated decay and lower rates of orthodontic treatment and fissure sealant application among children with a migration background. Although oral hygiene behaviours were not primary outcomes of this review, differences in these behaviours between migrant and non-migrant populations provide important context for interpreting observed disparities in clinical outcomes. Lieske et al. [12] further demonstrated that middle-aged migrant adults had poorer oral hygiene (higher Approximal Plaque Index scores), more untreated caries, and were more likely to seek care only in response to pain, whereas non-migrants utilized preventive services more regularly. Moreover, qualitative studies such as those by Van Steenkiste [39] revealed that Turkish parents often held negative perceptions of dentists, feeling inadequately informed or even judged during consultations, sentiments that likely deter regular dental visits. Oral hygiene behaviours (e.g., toothbrushing frequency) were not predefined outcomes of this review but are discussed to contextualize observed disparities in oral health status and dental service utilization. Knopf et al. [40] showed that children from migrant families were more likely to brush their teeth less frequently and attend fewer check-ups than their non-migrant peers, and Kühnisch et al. [41] found similar patterns in younger children, with a clear deficit in the application of fissure sealants among migrants. These examples demonstrate how both clinical and behavioural indicators contribute to a consistent pattern of disadvantage.

Notably, several studies utilized the PICO framework, enabling direct comparisons across migrant and non-migrant groups and revealing the cumulative effects of structural barriers. For example, Brzoska et al. [42] and Erdsiek et al. [43], using large national datasets, reported that migrants, especially those with lower socioeconomic status, had significantly lower odds of attending annual dental check-ups, even after adjusting for confounders like age, gender, and insurance status.

### Risk of bias assessment

The risk of bias for the included cross-sectional studies was assessed using the AXIS tool, and the findings are summarized in Tables 2, 3 and 4. Overall, the methodological quality was moderate to high across studies. All studies clearly stated their objectives and employed appropriate designs for evaluating oral health disparities between migrant and non-migrant populations in Germany. However, only a minority of studies provided justification for sample size (20%) or adequately addressed potential non-response bias (13.3%). While the majority used validated measures and presented consistent results, some relied on convenience sampling or self-reported data, which may introduce selection and recall bias. Ethical approval was inconsistently reported (26.6%).

**Table 2:**
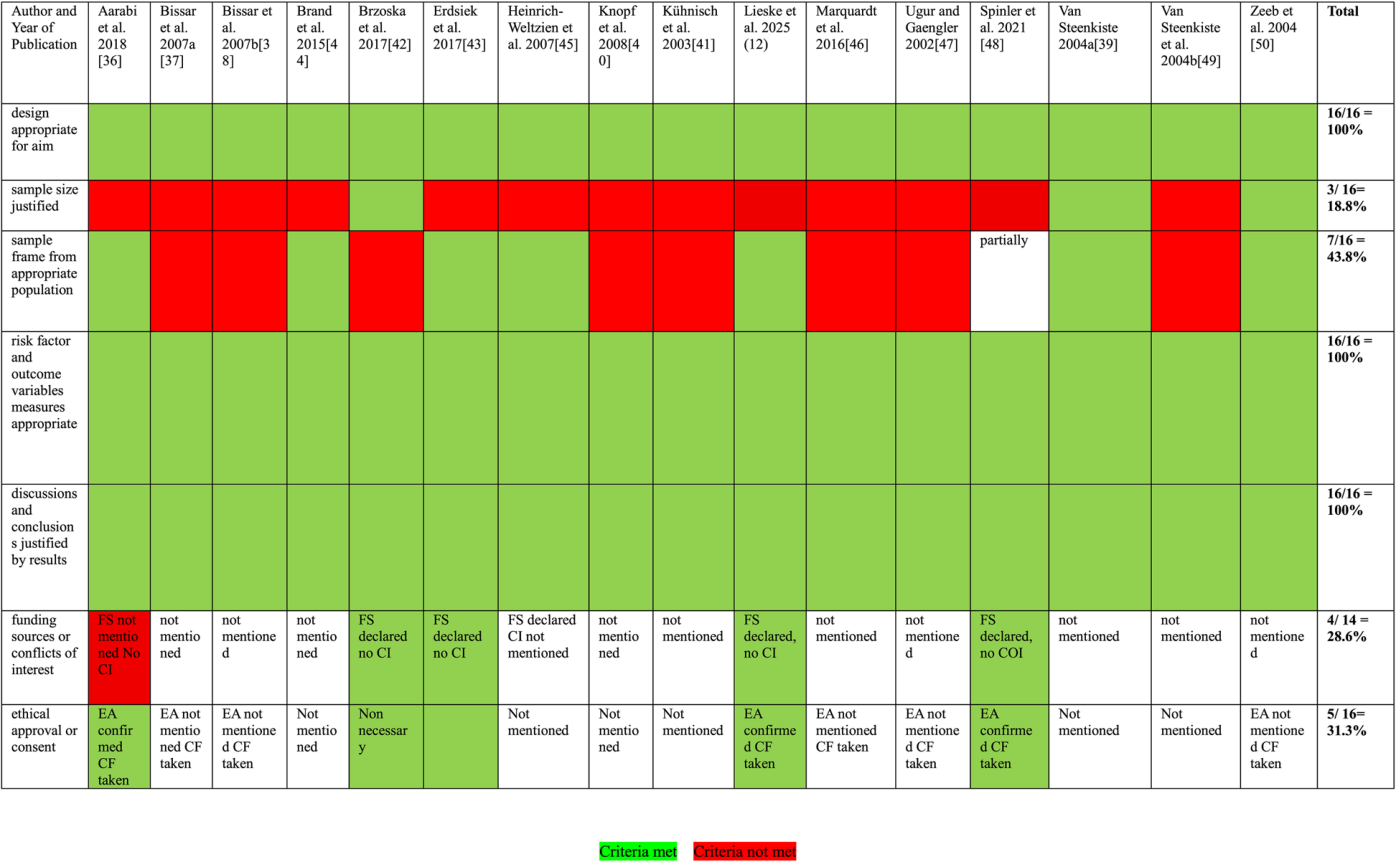
Critical appraisal using AXIS tool: quality of study design [12, 36–50]

**Table 3:**
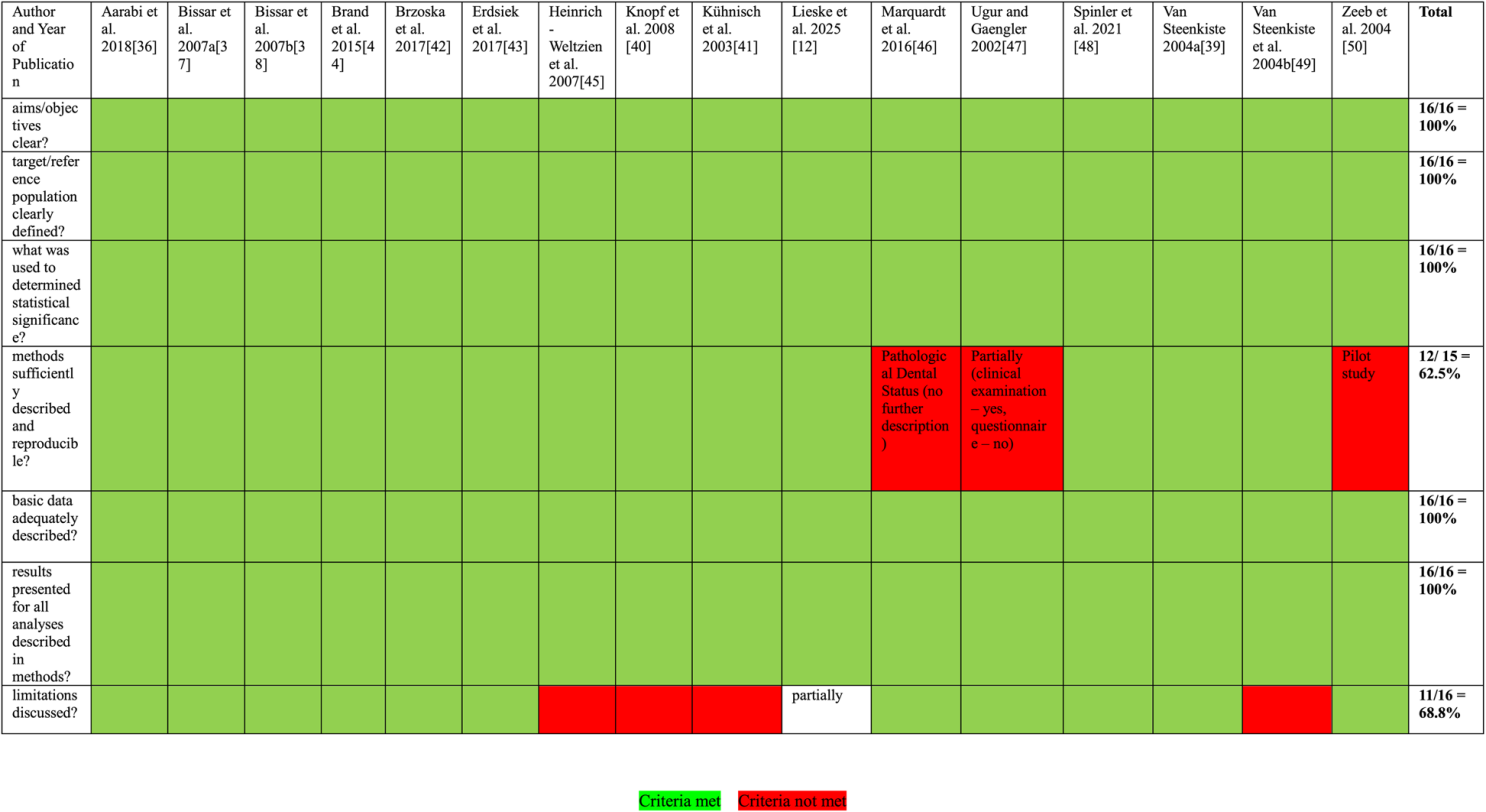
Critical appraisal using AXIS tool: quality of reporting [12, 36–50]

**Table 4:**
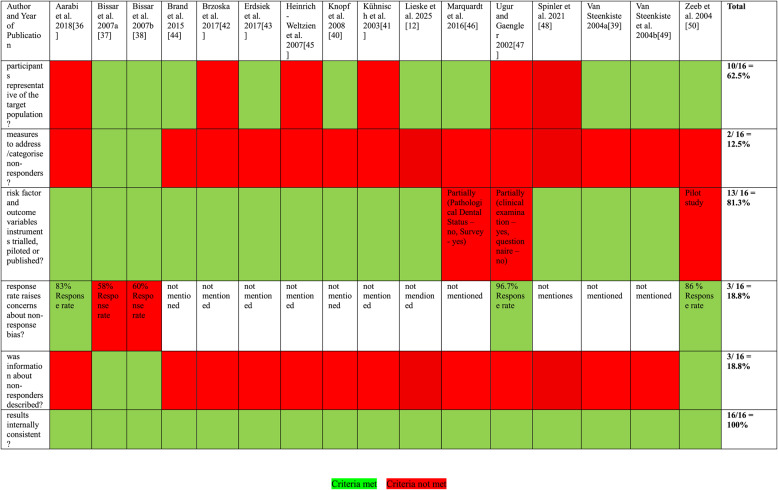
Critical appraisal using AXIS tool: risk of bias [12, 36–50]

### Certainty of evidence

The certainty of evidence, assessed using the GRADE approach (Table 5), ranged from low to moderate. While outcomes such as DMFT, missing teeth, and dental service utilization received moderate certainty ratings, others like untreated and filled teeth were rated with low certainty due to heterogeneity, reliance on self-reported data, and methodological limitations. These findings underscore the need for cautious interpretation of the results.

**Table 5:** Certainty of evidence using GRADE

Outcome	Number of Studies	Study Design	Study Limitation (Risk of Bias)	Inconsistency of Results	Indirectness of Evidence	Imprecision	Publication Bias	Number of Participants	Effect Size	Certainty of Evidence
Dental Caries index (DMFT)	7 (Aarabi et al., Bissar et al. 2007a & 2007b, Heinrich-Weltzien et al., Kühnisch et al., Lieske et al.)	Cross-sectional and RCT	Moderate	Low	None	Low	Possible	~ 10,000 +	SMD = 0.40 [95% CI: 0.31–0.49] (moderate)	⊕ ⊕ ⊕ ○ Moderate
Untreated Caries (D Component)	6 (Aarabi et al., Bissar et al. 2007a & 2007b, Heinrich-Weltzien et al., Kühnisch et al., Lieske et al.)	Cross-sectional and RCT	High	High	None	High	Possible	~ 7,000 +	SMD = 0.28 [95% CI: −0.09–0.65] (small to moderate)	⊕ ⊕ ○○ Low
Missing Teeth (M Component)	6 (Aarabi et al., Bissar et al. 2007a & 2007b, Heinrich-Weltzien et al., Kühnisch et al., Lieske et al.)	Cross-sectional and RCT	Moderate	Low	None	Moderate	Possible	~ 3,000 +	SMD = 0.17 [95% CI: 0.08–0.26] (small)	⊕ ⊕ ⊕ ○ Moderate
Filled Teeth (F Component)	6 (Aarabi et al., Bissar et al. 2007a & 2007b, Heinrich-Weltzien et al., Kühnisch et al., Lieske et al.)	Cross-sectional and RCT	High	High	None	High	Possible	~ 3,000 +	SMD = 0.02 [95% CI: −0.60–0.64] (negligible)	⊕ ⊕ ○○ Low
Periodontal Health	2 (Aarabi et al., Lieske et al.)	Cross-sectional and RCT	Moderate to High	Moderate	None	High	Possible	~ 2,000 +	Higher Gingival Inflammation & PBI Scores	⊕ ⊕ ⊕ ○ Moderate
Dental Service Utilization	5 (Aarabi et al., Lieske et al., Knopf et al., Kühnisch et al.r et al. 2021)	Cross-sectional and RCT	Low to Moderate	Low	None	Low	Possible	~ 60,000 +	OR ~ 0.64–0.67 for migrants vs non-migrants	⊕ ⊕ ⊕ ○ Moderate
Oral Health Literacy	3 (Aarabi et al., Lieske et al.; Spinler et al. 2021)	Cross-sectional and RCT	Low	Moderate	None	Moderate	Possible	~ 2,000 +	OHLP Score lower in migrants (~ 49 vs. 61)	⊕ ⊕ ⊕ ○ Moderate

The majority of included studies were observational in nature, predominantly cross-sectional. One cluster-randomized controlled trial (Lieske et al. [12]) was included; however, only its baseline, pre-randomization data were used, as these reflect observational comparisons between migrant and non-migrant participants. Because no intervention effects were analyzed, the study was appraised as an observational study for the purposes of evidence synthesis and GRADE assessment

Overall certainty of evidence remained low to moderate across outcomes, consistent with the predominance of observational designs. Certainty was upgraded in cases of large and consistent effect sizes or when results were supported by representative datasets (e.g., dental service utilization). Nevertheless, factors such as heterogeneity, reliance on self-reported measures, and unclear risk of bias in several domains prevented any outcome from reaching high certainty. Most findings were therefore rated as moderate or low certainty, reflecting a cautious but meaningful level of confidence in the observed disparities in oral health between migrant and non-migrant populations in Germany

### Meta-analysis

The meta-analysis pooled observational comparisons between migrant and non-migrant populations. No intervention effects were evaluated.

**Fig. 2:**
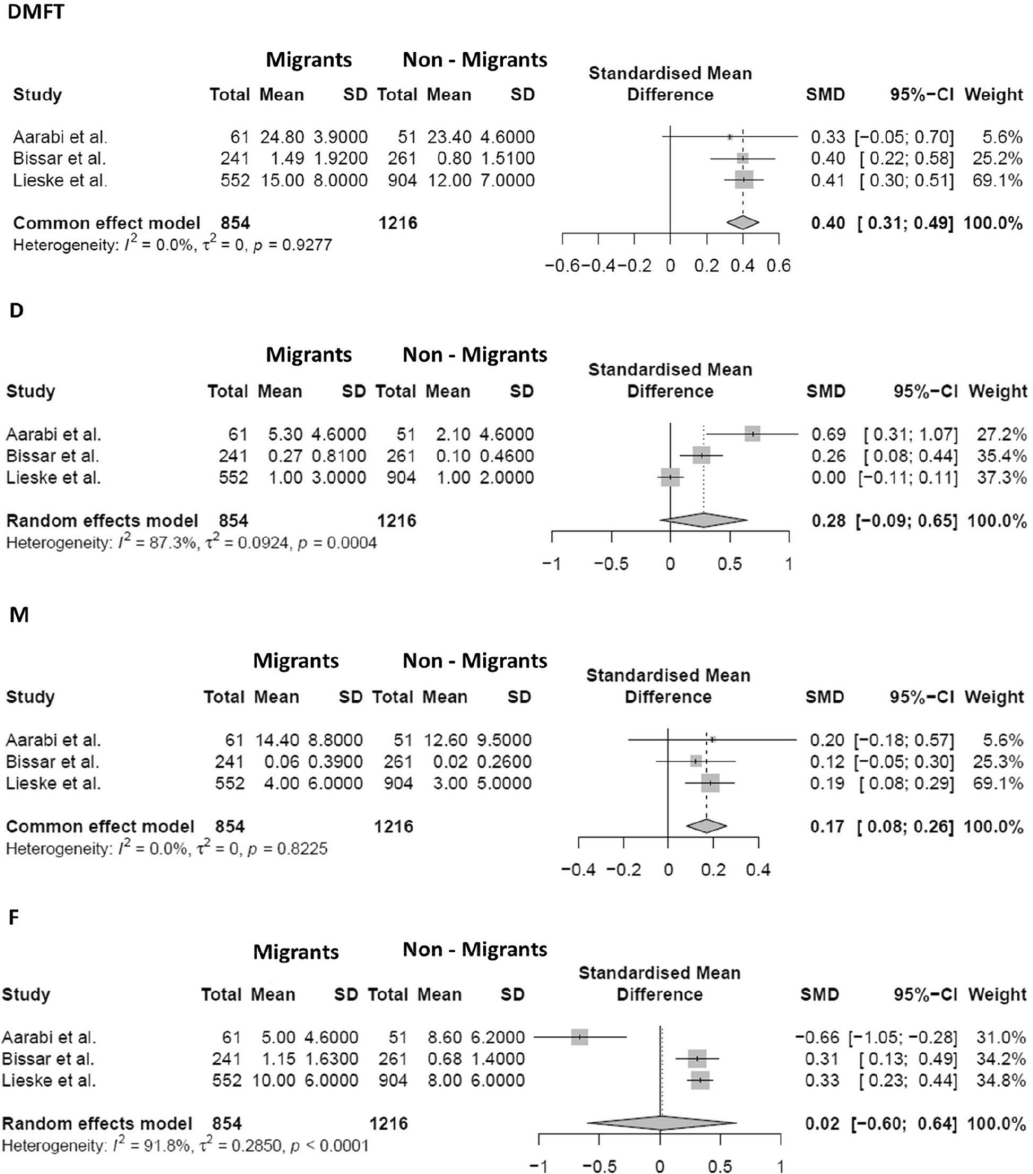
Forest Plots showing the Standard Mean Deviation (SMD) for the total DMFT and each separate parameter (D, M, F) between migrant group

Due to significant variability in the methodologies used to quantitatively assess oral health status across studies, a meta-analysis was feasible only for the DMFT index (Decayed, Missing, Filled Teeth). The DMFT is a well-established and extensively validated metric that provides a standardized assessment of an individual’s overall dental health. The meta-analysis findings (Fig. 2) across the included studies (Aarabi et al., Bissar et al., and Lieske et al.) [12, 36, 37] revealed variability in the magnitude of differences between migrant and non-migrant populations across studies. It furthermore revealed a statistically significant difference between non-migrant and migrant group in DMFT and M components (DMFT: SMD = 0.40; 95% CI: [0.31, 0.49]; I^2^ = 0%, *p* < 0.05; M: SMD = 0.17; 95% CI: [0.08, 0.26]; I^2^ = 0%, *p* < 0.05) with no observed heterogeneity, which indicated that the migrant group had significantly more missing teeth (M), resulting in a higher overall DMFT score compared with the non-migrant group. In contrast, no statistically significant difference was found between the two groups in D and F components (D: SMD = 0.28; 95% CI: [−0.09, 0.65]; I^2^ = 87.3%, *p* > 0.05; F: SMD = 0.02; 95% CI: [−0.06, 0.64]; I^2^ = 91.8%, *p* > 0.05), when high heterogeneities were obtained. This demonstrated that the number of decayed and filled teeth are similar in both migrant and non-migrant population.

### Sensitivity analysis

**Fig. 3:**
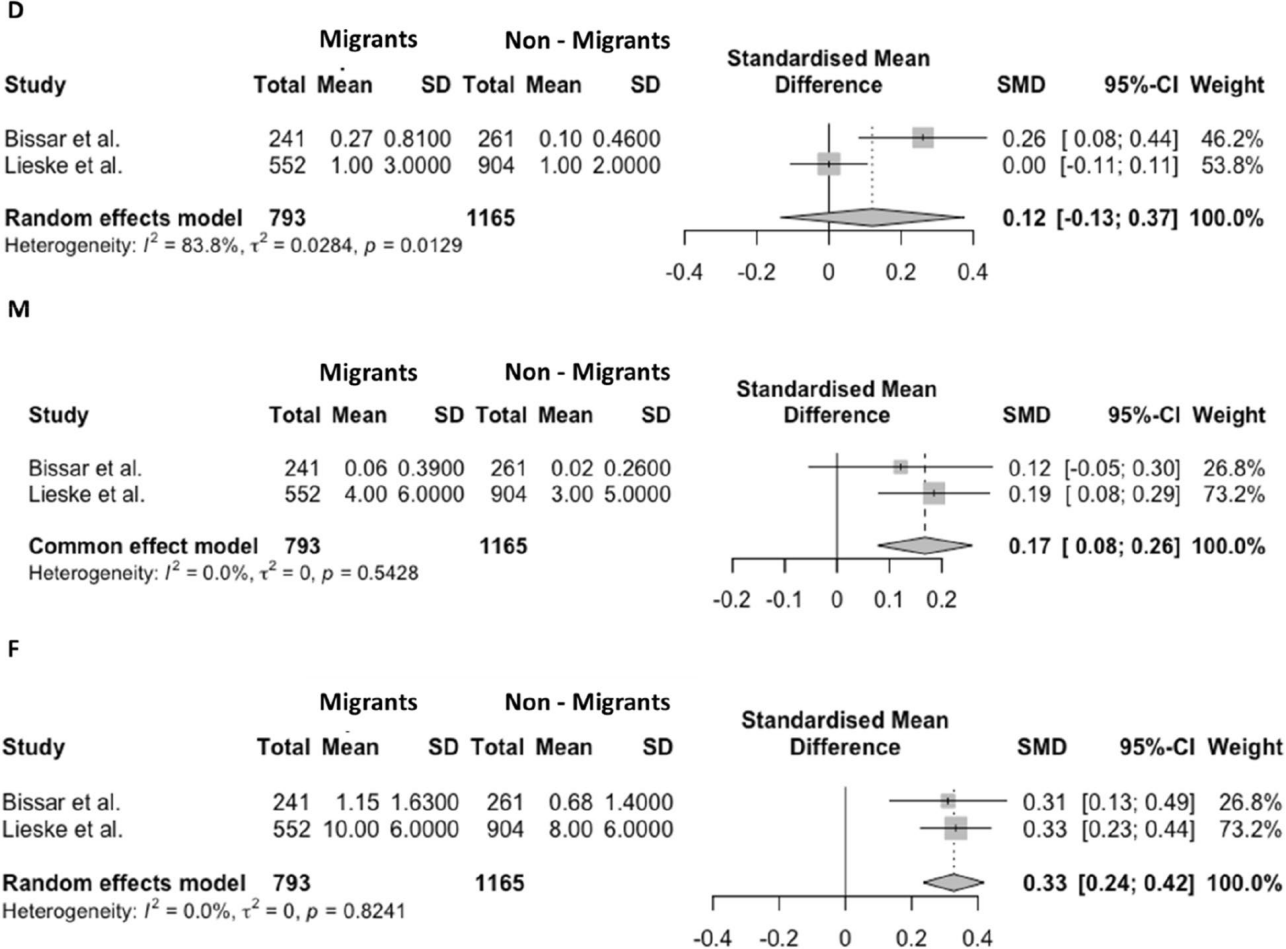
Sensitivity analysis: Forest Plots showing the SMD for D,M, F parameters when Aarabi et al. [36] was excluded

Considerable high heterogeneity was established among the trials from the results of D (I^2^ = 87.3%) and F (I^2^ = 91.8%) components. Since Aarabi et al. [36] presented data with most different age group (60 +) and socioeconomic factors compared to other two studies. To explain the potential source of high heterogeneity from these two outcomes and to assess the robustness of the results in the meta-analysis as well, outcomes were calculated again when Aarabi et al. [36] was excluded (Fig. 3). The results showed no statistically significant differences between the two groups in D component (SMD = 0.12; 95% CI: [−0.13, 0.37]; I^2^ = 83.8%, *p* > 0.05) with high heterogeneities. Conversely, in the F components analysis, significant higher value was found in the migrant group (SMD = 0.33; 95% CI: [0.24, 0.42]; I^2^ = 0%, *p* < 0.05) with no observed heterogeneity. Due to these high heterogeneities and seemingly reversed results in the F-component, after Aarabi et al. [36] exclusion, the reliability of the findings (D and F) is limited and should be interpreted with caution.

## Discussion

The findings from this systematic review reinforce the growing body of evidence indicating significant oral health disparities between migrant and native populations in Germany. Across the included studies, consistent patterns emerged, demonstrating that migrants experience higher rates of untreated dental caries, poorer periodontal health, and lower engagement with preventive dental care services. These factors disproportionately affect migrant populations and may help explain the consistently poorer oral health outcomes observed compared with non-migrants.

Germany’s migrant population is historically and socially heterogeneous, shaped by successive waves of migration since the post–World War II labour shortage. Early recruitment of so-called “guest workers” from Southern Europe (notably Italy and Greece) and later Turkey during the 1950s–1970s was followed by the immigration of ethnic German late resettlers from Eastern Europe after the collapse of the Soviet Union, and more recently by refugees and migrants from low- and middle-income countries driven by war, political instability and economic hardship. This heterogeneity is reflected in the studies included in this review, which encompass first- and second-generation migrants, children of former labour migrants, asylum seekers and long-settled populations, underscoring that observed oral health disparities cannot be attributed to a single migrant profile but rather to cumulative structural and social disadvantage across migration histories.

### Oral health status

The Decayed, Missing, and Filled Teeth (DMFT) index remains one of the most reliable indicators of dental health status. The studies included in this review consistently reported significantly higher DMFT scores among migrant populations, primarily attributable to higher missing-teeth (M) components, with more variable findings for decayed (D) teeth and generally lower or comparable filled-teeth (F) components.

The meta-analysis confirmed significantly poorer oral health among migrant groups, with higher overall DMFT and missing-teeth (M) (I^2^ = 0.0%) scores. In contrast, findings for untreated decay (D) (I^2^ = 87.3%) and filled teeth (F) (I^2^ = 91.8%), varied considerably across studies. Bissar et al. [37] and Aarabi et al. [36] reported higher levels of untreated caries among individuals with a migration background, whereas Lieske et al. [12] observed little difference between groups. Similarly, the lower or comparable filled-teeth component among the migrant population may indicate reduced access to restorative dental care. Aarabi et al. [36], for example, reported substantially lower restoration indices among migrant population (44%) compared with non-migrant population (79%). Research by Bissar et al. [38] and Kühnisch et al. [41] revealed higher levels of untreated caries and fewer restorations among migrant children compared to non-migrant children in Germany. Similar results were reported in Austrian research [51] linking a higher prevalence of dental caries in children with a migration background to a lower parental education and socioeconomic disadvantages.

The observed heterogeneity likely reflects the considerable diversity of the included study population and its migration histories. Aarabi et al. investigated elderly first-generation migrants who may have experienced lifelong barriers to dental care [36], whereas Bissar et al. [37] focused on schoolchildren and Lieske et al. [12] examined middle-aged adults with more heterogeneous migration backgrounds. Variability in recruitment settings and definitions of migration background may have further contributed to inconsistent findings, particularly regarding restorative treatment.

Beyond dental caries, migrant populations also exhibited poorer periodontal health than non-migrant populations. Studies included in this review reported higher levels of gingival inflammation, plaque accumulation and periodontal disease among participants with migration background, findings that were associated with lower utilization of preventive services and reduced access to professional dental care [36, 47]. Similar patterns have been reported internationally, including studies from Spain [52], suggesting that socioeconomic disadvantage, reduced preventive attendance and barriers to accessing regular dental care may contribute to poorer periodontal outcomes across migrant populations.

### Oral health literacy and dental service utilization

Lower oral health literacy and reduced utilization of preventive dental services may further contribute to the poorer oral health outcomes observed among migrants. Consistent with the review by Lauritano et al. [9], migrant populations were more likely to report lower health literacy, reduced preventive attendance and less frequent access to dental care. Language and cultural barriers were also identified as important factors affecting healthcare navigation and communication with dental providers [39, 47, 53], resulting in inequal health outcomes.

Large population-based studies further demonstrated that individuals from migrant backgrounds had significantly lower odds of attending regular preventive dental check-ups than non-migrants, despite adjustment for socioeconomic and insurance-related factors [42, 43]. These findings suggest that barriers to dental care extend beyond formal insurance entitlement and continue to be shaped by socioeconomic disadvantages and structural inequalities. Migrant populations are disproportionately represented in lower-income groups, which may limit access to out-of-pocket dental services and more comprehensive private insurance coverage. This association between financial constraints and reduced dental service utilization has been observed in other European countries, including the United Kingdom and Italy [54, 55]. Particularly vulnerable groups, such as asylum seekers, showed markedly reduced access to dental care, with Freiberg et al. [56], reporting substantially lower utilization of both general and preventive dental services compared with the general population.

Despite Germany’s universal healthcare system, these findings indicate that systemic barriers, including insurance-related restrictions, lack of culturally competent providers, and limited community outreach, continue to hinder equitable dental care access for migrant populations.

#### Strengths and Limitations

This review provides a comprehensive synthesis of the available evidence on oral health disparities between migrant and non-migrant populations in Germany. A major strength is the inclusion of both quantitative and contextual evidence, allowing clinical findings to be interpreted alongside structural, social and behavioural determinants of oral health. The use of a registered protocol, PRISMA-guided methodology, standardized quality assessment and GRADE evaluation further strengthens the transparency and methodological rigor of the review. Nevertheless, several limitations should be considered when interpreting the findings. Most included studies were observational and predominantly cross-sectional, limiting causal inference. In addition, substantial heterogeneity existed across studies regarding study populations, definitions of migration background, outcome measures and assessment methods. While some studies used standardized clinical indicators such as DMFT, others relied partly on self-reported measures, increasing the risk of measurement and recall bias. Selection bias and insufficient reporting of non-response were also common limitations.

The certainty of evidence ranged from low to moderate across outcomes. Evidence for DMFT scores, missing teeth, periodontal health and dental service utilization was generally more consistent and therefore rated as moderate certainty. In contrast, findings related to untreated and filled teeth showed considerable heterogeneity and were rated with lower certainty. Furthermore, migrants were frequently analysed as a single population despite considerable cultural, linguistic and socioeconomic diversity, which may obscure important subgroup-specific differences.

Despite these limitations, the evidence consistently indicates that migrant populations in Germany experience poorer oral health outcomes and lower utilization of preventive dental services compared with non-migrants. Future research should prioritize longitudinal and intervention-based studies, representative sampling strategies, and standardized outcome reporting to improve comparability and strengthen the evidence base.

## Conclusions

This systematic review demonstrates consistent and concerning oral health inequalities between individuals with a migration background and non-migrant populations in Germany. Migrant populations showed poorer oral health outcomes, including higher DMFT scores, poorer periodontal health and lower utilization of preventive dental services. These disparities appear to be influenced not only by socioeconomic disadvantages, but also by structural barriers, limited oral health literacy and language- or culture-related challenges in accessing dental care.

Despite Germany’s broad statutory health insurance coverage, equitable access to preventive and restorative dental care remains insufficient for many population groups with a migration background. The collective evidence highlights the pressing need for culturally sensitive, linguistically appropriate, and structurally inclusive healthcare strategies that address the specific barriers faced by migrant communities. Future research should prioritize longitudinal and intervention-based studies using standardized outcome measures and more differentiated analyses of specific subgroups to strengthen the evidence base and support more equitable oral healthcare in Germany.

## Supplementary Information

Supplementary Material 1.

## Data Availability

The data that support the findings of this study are available on request from the corresponding author. The data are not publicly available due to privacy or ethical restrictions.
