# Supplementary material for: Oral health in migrant population of Germany: a systematic review and meta-analysis
Source: BMC Public Health. 2026 Jun 8;26:1824. doi: 10.1186/s12889-026-27927-8 (PMC13244935; doi:10.1186/s12889-026-27927-8)
Supplement: Supplementary file 1 — Supplementary Material 1. [file 12889_2026_27927_MOESM1_ESM.docx]

Appendix

**Tab. 1: Extraction Table for included Studies**

| **Study and Study Type** | **Language** | **Population** | **Group 1** | **Group 2** | **Endpoints** | **Results** | **Conclusions by the authors** |
| --- | --- | --- | --- | --- | --- | --- | --- |
| **Aarabi et al. 2017**  **Cross-Sectional Observational Study**  **PMID:**  28277023 | English | N= 112  **Inclusion Criteria:**  age 60 or above, residents of Hamburg metropolitan area, first-time patients at the dental practice, German language proficiency and ability to consent  **Age:**  =/> 60 years  **Time frame:**  November 2012 - March 2014  **Follow-up:**  none | Migrants  N=61  (Immigrants born in a country other than Germany) | Non-Migrants  N=51  (German natives born in Germany) | Primary:  DMFT  (Decayed, Missing, Filled Permanent Teeth) | - Higher DMFT, D in migrants - lower F in migrants - poorer oral hygiene in migrants - p < 0.001 | *Elderly migrants have poorer oral health and thus a higher need*  *of treatment than non-migrants. Likely causes, were*  *less effective oral hygiene and lower utilization of dental care services by migrants.*  *It seems*  *that specific prevention programs targeting migrants are warranted to improve oral*  *health in this disadvantaged group.* |
|  |  |  |  |  | Secondary:  Restoration index |  |  |
|  |  |  |  |  | API in %  (Approximal Plaque Index) |  |  |
|  |  |  |  |  | PBI in %  (Papilla Bleeding Index) |  |  |
|  |  |  |  |  | PBI<40% = good oral hygiene, >40% fair to poor oral hygiene |  |  |
|  |  |  |  |  | face-to-face interview, 18 questions corresponding to the German Oral Health Survey (DMS) IV (Micheelis and Schiffner 2006).  1) Sociographic status (age, gender, family status, religion) and socioeconomic status (education, monthly household net income, professional status).  2) Utilization of dental care services  3) Oral hygiene behaviour and information on difficulties receiving dental treatment | - Average time of residency in Germany: 37 y - Education among non-migrants significantly higher (university degree) compared to migrants (low degree or <10 y school) - Significantly more non-migrants have regular visits with a dentist (82.4% vs. 55.7%), similar for deep cleaning - More non-migrants used additional oral hygiene aside from tooth brush and paste. - 2/3 migrants reported difficulties in receiving dental care: costs and language barriers as main reasons - 78.4% of non-migrants had no difficulties |  |

| **Study Type** | **Language** | **Population** | **Group 1** | **Group 2** | **Endpoints** | **Results** | **Conclusions by the authors** |
| --- | --- | --- | --- | --- | --- | --- | --- |
| **Bissar et al. 2007a**  **Cross-Sectional Observational Study**  **PMID:**  17683326 | English | N= 502  **Inclusion Criteria:**  Children (5th, 6th, 7th grade) at 5 schools in areas of disadvantaged socio-economic status, in Heidelberg, with high proportions of immigrant children.  **Age:**  11-13 years  **Time frame:**  May - June 2004  **Follow-up:**  none | Migrants  N= 241  (children with an immigrant background: whose parents (one or both) were born outside of Germany) | Non-Migrants  N=261  (without immigrant background: whose parents were born in Germany) | Primary:  carious, missing, filled teeth (DMFT) and fissure sealants | - Higher DMFT - higher D/M - lower F and fissure sealants in migrants - significantly higher unmet dental treatment needs (DT/DFT) (p < 0.01) - lower orthodontic treatment uptake in migrants (p < 0.01) | *Inequalities in oral health between the immigrant and the German schoolchildren still exist and action needs to be taken quickly. The biggest part of the responsibility is undoubtedly related to the deficient*  *oral health knowledge of the parents and the*  *children. The immigrants have not yet been enabled to demand all preventive and therapeutic measures, which they are entitled to.* |
|  |  |  |  |  | Secondary:  Unmet Dental Treatment Needs (DT/DFT) |  |  |
|  |  |  |  |  | current/ previous orthodontic treatment (fixed or removable) |  |  |

| **Study Type** | **Language** | **Population** | **Group 1** | **Group 2 and 3** | **Endpoints** | **Results** | **Conclusions by the authors** |
| --- | --- | --- | --- | --- | --- | --- | --- |
| **Bissar et al. 2007b**  **Cross-Sectional Observational Study**  **PMID:**  18704289 | English | N=570  **Inclusion Criteria:**  Schoolchildren (5th, 6th, 7th grade) in 5 schools in Heidelberg with high proportions of immigrant pupils, living in socially disadvantaged areas.  **Age:**  11-14 years, Average: 12.3 y  **Time frame:**  May - June 2004  **Follow-up:**  none | Non-migrants (Gr 1)  N=288  (without immigrant experience: children and parents born in Germany) | Migrants (Gr 2)  N=191  (indirect immigrant experience: children born in Germany, whose parents (one or both) were born outside of Germany=  Migrants (Gr 3)  (N=91)  (immigrant experience: children and parents born outside of Germany) | Primary:  DMFT (Mean Values and Standard Deviations) | - Group 2 and 3 had significantly more caries experience compared to Group 1 (p<0.05) - Diseased Teeth (D) was significantly higher in Group 2 (p<0.05) and Group 3 (p<0.05) compared to Group 1 - Significant difference of caries-free dentition between Group 1 and 2 (p<0.01) and 1 and 3 (p<0.01) - Immigrant children born in Germany had lowest proportion of caries-free dentition | *Immigration background still has a negative impact on oral health of children in Germany. Thus, there is a need to develop effective and appropriate public health policies and community-based oral health*  *care programmes focusing to improve the oral health behaviour and attitudes of immigrant parents and children. Close*  *collaboration with key persons in societies of ethnic minority,*  *organisation of dental health education meetings, and distribution of educational materials offered in the languages of these minorities are therefore very important basic steps. Preventive concrete programmes comprising i. e. the application*  *of fluorides, the widespread use of fissure sealants must be*  *also offered in parallel for these children.* |
|  |  |  |  |  | Secondary:  DMFT male / female |  |  |
|  |  |  |  |  | Caries-free dentition in % according to age |  |  |
|  |  |  |  |  |  |  |  |

| **Study Type** | **Language** | **Population** | **Group 1** | **Group 2** | **Endpoints** | **Results** | **Conclusions by the authors** |
| --- | --- | --- | --- | --- | --- | --- | --- |
| **Brand et al. 2015**  **Narrative Review**  **PMID:**  25861044 | German | N=22.048  **Inclusion Criteria:**  Data  from the "Gesundheit in  Deutschland Aktuell" survey from 2010 conducted by the RKI.  landline phone, permanent residence, sufficient German language skills  **Age:**  =/>18 years  **Time frame:**  2010  **Follow-up:**  none | Migrations  N= 3004 | Non-Migrants  N= 19.044 | Participation in a dental health prevention check-up during the last 12 months (age, gender): 95%CI compared with Group 1. | - lower participation in dental check-ups in 2009/2010 by approx. 10 percentage points in migrations (male and female over all ages) - participating in the dental care examinations, the factors that are influenced by migration status, gender and social class add up to approx. 35 percentage points (men with a migration background and low social 53.0%, 95% CI: 48.9-57.1   women without a migration background and with high social class affiliation: 88%, 95% CI: 86.4-89.5). | *Among adults with a migrant background,*  *we found lower participation in general*  *health check-ups, oral health check-ups, cancer screening programs and influenza vaccination.* |
|  |  |  |  |  | Participation in a dental health prevention check-up during the last 12 months (gender, social status): 95%CI compared with Group 1. |  |  |
|  |  |  |  |  |  |  |  |

| **Study Type** | **Language** | **Population** | **Group 1** | **Group 2** | **Endpoints** | **Results** | **Conclusions by the authors** |
| --- | --- | --- | --- | --- | --- | --- | --- |
| **Brzoska et al. 2017**  **Cross-Sectional Analysis**  **PMID:**  28856132 | English | N= 41,220  **Inclusion Criteria:**  telephone surveys (“German Health Update 2009” and “German  Health Update 2010”), carried out between July 2008 and July  2010 by the Robert Koch Institute.  In Germany residents, who lived in a private household with landline telephone.  **Age:**  =/> 18 years  **Time frame:**  July 2008 and July 2010  **Follow-up:**  none | Migrants  N~6,183  (individuals who had migrated to Germany or whose parents (at least one) had migrated to Germany.) | Non-migrants  N~35,037 | SES based on educational background, occupational status and net equivalent income (low, middle, high) | - Migrants were on average younger than non-migrants - Group 1 about 36% lower chance of utilizing regular dental check-ups than Group 2 (OR = 0.64; AME = −0.081) --> influence of predisposing and enabling factors (OR = 0.69; AME = −0.065): younger age, being male, lower SES, a non-statutory health insurance, and poor social support - Generally: individuals who did not live in a relationship, in the Western part of Germany, in an urban setting had lower chance of regular dental checkups - Older migrants were more likely to utilize dental prevention than younger respondents. | *Migrants utilize dental checkups*  *less frequently than non-migrants. Differences are only partially*  *explained by the different enabling and predisposing factors which we were able to take into account as a multivariable analysis adjusting for these factors shows.* |
|  |  |  |  |  | Health insurance (statutory, private/other) |  |  |
|  |  |  |  |  | marital status (living with or without a partner) |  |  |
|  |  |  |  |  | place of residence (former West or East Germany) and type of residence (urban, rural) |  |  |
|  |  |  |  |  | social support based on Oslo-3- Social Scale (poor, moderate or strong) |  |  |

| **Study Type** | **Language** | **Population** | **Group 1** | **Group 2** | **Endpoints** | **Results** | **Conclusions by the authors** |
| --- | --- | --- | --- | --- | --- | --- | --- |
| **Erdsiek et al. 2017**  **Cross-Sectional Survey Study**  **PMID:**  28526074 | English | N= 138,500  **Inclusion Criteria:**  ‘German Health Update 2010’ conducted by the Robert  Koch Institute.  German Residents, German language proficiency, landline telephone, private household  **Age:**  = / > 18 years  **Time frame:**  September 2009 to July 2010  **Follow-up:**  none | Migrants  N= 21,741  (migrated themselves or at least one parent migrated to Germany)  (N= 21,741 =15.7%) | Non-Migrants  N ~ 116,759 | age | - migrants were significantly younger - Utilization of dental prevention overall lower among migrants (72.6%) than non-migrants (79.8%), corresponding to a crude odds ratio (OR) of 0.67. - Overall: Chances for utilization increased consistently with higher socioeconomic status. Female, living in co-habitation enhanced chance of utilizing dental check-ups - Living in West Germany reduced chance of utilization. | *The utilization of annual dental check-ups in*  *Germany is lower among adult migrants than among non-migrants, even after taking into account several*  *demographic, socioeconomic and contextual factors.*  *Our findings highlight the necessity of culturally sensitive and targeted services in the dental care setting. This includes*  *communication on the system level (in terms of providing information on existing services and structures)*  *as well as on the provider level (in terms of patient-*  *provider interaction), the design and delivery of services (in terms of patient approach, waiting times and*  *opening hours) as well as patient education to enhance health literacy.* |
|  |  |  |  |  | SES (high, middle, low) |  |  |
|  |  |  |  |  | place of residency (former West or East Germany, urban or rural) |  |  |
|  |  |  |  |  | co-habitation (living with or without partner) |  |  |
|  |  |  |  |  | Type of Health Insurance (statutory or private/ other) |  |  |
|  |  |  |  |  | utilization of dental check-ups in 12 months prior to the interview. |  |  |

| **Study Type** | **Language** | | **Population** | | **Group 1** | **Group 2 and 3** | | **Endpoints** | | **Results** | | | **Conclusions by the authors** |  |
| --- | --- | --- | --- | --- | --- | --- | --- | --- | --- | --- | --- | --- | --- | --- |
| **Heinrich-Weltzien et al. 2007**  **Cross-Sectional Observational Study**  **PMID:**  17405082 | German | | N= 1.736  **Inclusion Criteria:**  Only Turkish OR German population. 12-15-year olds who underwent dental prophylaxis programme during primary school (1993) AND 12-15-year olds who additionally underwent group prophylaxis during kindergarten (2003) --> as part of a systematic prophylaxis programme. 90% of Turkish students and 30% of Germans went to Hauptschule, 30% of Germans went to Gymnasium.  **Age:**  12-15 years  **Time frame:**  1993-2003  **Follow-up:**  none | | Migrants  N= NA*  (Turkish students in Hauptschule) | Non-Migrants (Gr 2)  N= NA  (German students in Hauptschule)  Non-Migrants (Gr 3)  N= NA  (German students in Gymnasium) | | population composition in % | | - 1993: 352 12-year-olds AND 402 15-year-olds - 2003: 505 12-year-olds AND 477 15-year-olds   12-year-olds:   - the proportion of caries-free in Group 3 increased by more than 40 % and in Group 2 by more than 20%. - caries-free in Group 1 remained almost unchanged between 1993 and 2003   15-year-olds:   - similar - highest increase in caries-free dentition was recorded by Group 3 (27%) - followed Group 2 (17%), while the proportion of Group 1 with caries-free dentition did not change in the 10-year period   2003 in 12-year-olds:   - 3 % of all Hauptschul-students had 70 % of the total caries infestation with more than 3 DMFT and 9 % of all Gymnasium-students had 76 % of the total caries infestation with more than 1 DMFT   2003 in 15-year-olds:   - 73 % of the total caries infestation (>3 DMFT) was accounted for by 32 % of Hauptschul-students and 84 % of the total caries infestation (>1 DMFT) was concentrated in 25% of Gymnasium-students. | | | *The Gymnasium-students had the lowest proportion of decayed teeth*  *(D %) and missing teeth (M %) and the highest proportion of filled teeth (F %). With almost equal distribution of the D and F components, however, the Turkish pupils in both age groups and at both points in time of the study had the highest proportion of M.*  *Hauptschul-students, for example, showed poorer dental health in both age groups when compared to Gymnasium-students.*  *In the group of Hauptschul-students, it was the Turkish pupils who did not benefit from the preventive measures. Neither among the 12- nor the 15-year-old Turkish Hauptschul-students the proportion of pupils with caries-free teeth changed*. |  |
|  |  |  |  |  |  |  |  | Primary  DMFT-Index | |  |  |  |  |  |
| * NA meaning non-applicable as there was no data available | | | | | | | | | | | | | | |
| **Study Type** | **Language** | **Population** | | **Group 1** | | | **Group 2** | | **Endpoints** | | **Results** | **Conclusions by the authors** | | |
| **Knopf et al. 2008**  **Cross-Sectional Survey**  **PMID:**  19043760 | German | N= 17.641  **Inclusion Criteria:**  Children living in Germany, whose parents are insured in either statutory or private insurance.  Survey was taken by parents about their children!  **Age:**  0-17 years  **Time frame:**  May 2003-May 2006  **Follow-up:**  none | | Migrants  N= NA*  (children with migration background) | | | Non-Migrants  N= NA  (children without migration background= | | infrequent tooth brushing** (<3y less than once a day, =/>3y less than twice a day).  infrequent dental check-ups (less than once a year).  caries-protective medication used in the 7 days prior to the interview (fluoride tablets, only 0-6 y olds).  infrequent tooth brushing (<3y less than once a day, =/>3y less than twice a day).  infrequent dental check-ups (less than once a year).  caries-protective medication (fluoride tablets, only 0-6 y olds). | | - Children with a migration background were significantly more likely to:  1. Brush teeth less frequently 2. Miss regular dental check-ups 3. Use fewer fluoride supplements  - Migration background and low socioeconomic status were strong predictors of inadequate oral hygiene behaviours. - Highlights the role of parental health behaviour and awareness in shaping children's oral health. | *Children from immigrant families exhibit poorer caries prevention behaviour: more likely to fall short of the recommended toothbrushing frequency, and according to self-report they go to dental check-ups less often and take fluoride tablets to a lesser extent than children without a migration background.*    *Suggests a need for early preventive interventions and education targeting migrant families.* | | |

* NA meaning non-applicable as there was no data available

**Oral hygiene behaviours (e.g. toothbrushing frequency) were not predefined outcomes of this review and are reported here as contextual variables to aid interpretation.

| **Study Type** | **Language** | **Population** | **Group 1** | **Group 2** | **Endpoints** | **Results** | **Conclusions by the authors** | |
| --- | --- | --- | --- | --- | --- | --- | --- | --- |
| **Kühnisch et al. 2003**  **Longitudinal Observational Study**  **PMID:**  12632318 | German | N=369  **Inclusion Criteria:**  School children (born in 1989) living in the area of Ennepe-Ruhr-Kreis, Germany  **Age:**  Mean 8.0-9.8 years (beginning to end of study)  **Time frame:**  1997-1999  **Follow-up:**  2 years | Migrants  N=69  (Children of Immigrant families) | Non-Migrants  N=300  (German Children) | Primary:  DMFT/S  Caries free dentition in %  Secondary:  Molars with Fissure Sealants (FS)  Fissure Sealants intact (intact: no missing material in fissure) in% | - Children from immigrant families had significantly higher DMFS scores (decayed, missing, filled surfaces) in both 1997 and 1999. - Higher caries prevalence on permanent molars and more initial caries in migrants. - Lower use of fissure sealants among migrant children. - Indicates lower engagement with preventive dental care in migrant populations. | | *The causal barriers must be societal and psychosocial factors, a traditionally*  *limited utilization of dental care measures, communication difficulties, negative experiences and misunderstandings during previous visits to the dentist, a low level of education*  *on the part of the parents and/*  *or a lack of insurance cover can be assumed.* |

| **Study Type** | **Language** | **Population** | **Group 1** | **Group 2** | **Endpoints** | **Results** | **Conclusions by the authors** |
| --- | --- | --- | --- | --- | --- | --- | --- |
| **Lieske et al. 2025**  **Cluster-randomized Controlled Trial (MuMi Study)**  **PMID:**  40066255 | English | N= 1456  Inclusion Criteria: Adults aged ≥18 years, residing in Hamburg, Germany, recruited from 40 dental practices.  Age: Mean 44y  Time frame: December 2019 – March 2021  Follow-up: none | Migrants  N=904 | Non-Migrants  N=552 | Primary:  DMFT (Decayed, Missing, Filled Teeth) | - OHLP-Score significantly higher in Group 2 (p<0.001) - API significantly higher in Group 1 (p<0.001) - Caries Restoration Degree significantly higher in Group 2 (p<0.001) - DMFT significantly higher in Group 2 (p<0.001) - D significantly higher in Group 2 (p<0.001) - M significantly higher in Group 2 (p<0.001) - F significantly higher in Group 2 (p<0.001) - Last dental visit in 12 m significantly higher in group 2; Reasons: - *Preventive reasons* significantly higher in group 2 - *Pain/ Dental Problem* significantly higher in group 1 - Last dental cleaning in 12 m significantly higher in group 2 - Brushing teeth* at least once per day significantly higher in group 2 - Use of floss/interdental brush at least weekly significantly higher in group 2 - Brushing duration ≥2 minutes significantly higher in group 2 | *Migrants had significantly lower oral health literacy, poorer oral hygiene (higher API values), and a more problem-oriented approach to dental care. Lower degree of caries restoration and lower utilization of professional dental cleanings suggest gaps in preventive care. Better oral health literacy was significantly associated with improved oral hygiene.* |
|  |  |  |  |  | Secondary:  Oral Health Literacy Profile (OHLP-Score) |  |  |
|  |  |  |  |  | API % (Approximal Plaque Index - Oral Hygiene Status) |  |  |
|  |  |  |  |  | Caries Restoration Degree |  |  |
|  |  |  |  |  | Dental Service Utilization |  |  |
|  |  |  |  |  | Oral Hygiene Behaviour |  |  |

*Oral hygiene behaviours (e.g. toothbrushing frequency) were not predefined outcomes of this review and are reported here as contextual variables to aid interpretation.

| **Study Type** | **Language** | **Population** | **Group 1** | **Group 2** | **Endpoints** | **Results** | **Conclusions by the authors** |
| --- | --- | --- | --- | --- | --- | --- | --- |
| **Marquardt et al. 2016**  **Cross-Sectional Pilot Study**  **PMID:**  26610271 | English | N=102  **Inclusion Criteria:**  Unaccompanied, underage Asylum-Seekers in Bielefeld, Germany.  **Age:**  12-18 years, mean 16.04  **Time frame:**  8th September 2011 to 3rd July 2014. Medical Check-up performed within 1-2 weeks after arrival in Germany.  **Follow-up:**  up to 25 months | Migrants  N=102  (Asylum Seekers) | Explorative Study no Comparison | sociodemographic variables (age, sex, country of origin, region of origin)  Pathological Dental Status | - male: 76.5% - female: 23.5%   Region of Origin:   - South Asia 37.0% - Sub-Saharan Africa 29.0% - West Asia 15.0% - North Africa 13.0% - 20% had pathological dental findings shortly after arrival - Higher prevalence of dental issues among adolescents from Sub-Saharan Africa (37.9%) and North Africa (30.8%) - No follow-up data on treatment or long-term oral health. | *Findings point to urgent dental treatment needs in newly arrived asylum-seeking minors.*  *Highlights the importance of early screening and access to status.* |

| **Study and Study Type** | **Language** | **Population** | **Group 1** | **Group 2** | **Endpoints** | **Results** | **Conclusions by the authors** |
| --- | --- | --- | --- | --- | --- | --- | --- |
| **Spinler et al. 2022**  **Qualitative Study**  **PMID:**  35692338 | English | **N= 9 Experts**  **Inclusion Criteria:**  Experts: Professional experience in oral health and migration, focus group: Turkish migration background, German or Turkish language skills, living in Germany  **Age:** ≥18 y  **Time frame:**  August- October 2018  **Follow-up:** None | Explorative Study no Intervention | Explorative Study no Comparison | Identified themes:  barriers to dental care (language, knowledge, perceived importance, health socialization, dentist-patient interaction); dental service use behaviour; suggestions for improving cultural sensitivity | - Turkish migrants often delay dental visits until pain occurs - major barriers include language, low oral health knowledge, socioeconomic status, cultural norms, fear, and poor patient-dentist communication - Experts and participants emphasized the need for culturally sensitive materials, multilingual communication, and more inclusive dental practices | *Migrants, especially older or less educated ones, face multiple interacting barriers to oral health care. Enhancing intercultural competence in dental practice, increasing awareness campaigns, and collecting migrant-specific health data are essential steps toward equal access.* |

| **Study Type** | **Language** | **Population** | **Group 1** | **Group 2** | **Endpoints** | **Results** | **Conclusions by the authors** |
| --- | --- | --- | --- | --- | --- | --- | --- |
| **Ugur and Gaengler 2002**  **Cross-Sectional Observational Study**  **PMID:**  12090264 | English | N= 532  **Inclusion Criteria:**  Turkish migrants (children and adults) residing in Witten, Germany, interviewed in Turkish or German.  **Age:**  =/>12 years  **Time frame:**  1997  **Follow-up:**  none | Migrants  N= 532  (Turkish Migrants) | Explorative Study no Comparison | Primary:  DMFT [Mean (SD)] split by age groups  Secondary:  regular/ irregular visits to the dentist (every year vs only if tooth problem occurs) split by age groups  Periodontal Health as GPMT (=/> 4mm means periodontitis) split by age groups  Level of education, German proficiency level, Country of dental care, nationality of dentist in Germany, preferred country for dental care, perceived condition of oral health | - DMFT scores increased with age, with a notable rise in missing teeth among older adults. - Irregular dental attendance was common—45% to 70% only visited a dentist when problems occurred; 3.6% had never visited a dentist. - Lower treatment levels compared to the general German population. - Periodontal disease prevalence was high, especially in adults over 25. - German language proficiency and education level were positively associated with regular dental visits. | *Preference for receiving care in Turkey or from Turkish-speaking dentists in Germany indicated cultural and linguistic preferences in healthcare behaviour.* |

| **Study Type** | **Language** | **Population** | **Group 1** | **Group 2** | **Endpoints** | **Results** | **Conclusions by the authors** |
| --- | --- | --- | --- | --- | --- | --- | --- |
| **Van Steenkiste 2004a**  **Cross-Sectional Survey**  **PMID:**  14994207 | German | N=705  **Inclusion Criteria:**  Turkish and German population living in Rems-Murr-Area (Germany), random sample of parents whose children enrolled in primary school.  **Age:**  **-**  **Time frame:**  March - September 2000  **Follow-up:**  none | Migrants  N~36= 5.1% Turkish, N~99= 14.1% another ethnicity  (Turkish parents: Turkish nationality OR speaking Turkish with their children.) | Non-Migrants  N~570 = 80.8%  (German parents: German nationality AND speaking German with their children.) | "Dental Belief Survey", 12 phrases voted with Likert Scale (1-5, "fully agree" - "do not agree at all") | - Turkish parents: total score: 44.16 - German patents: total score: 50.91 - Overall differences (p<0.001) - Turkish parents showed more negative perceptions of dentists, particularly regarding communication and feeling judged - Appointment difficulties were more frequently reported by Turkish parents - Knowledge gaps were evident in understanding insurance co-payments and coverage - Language barriers were less reported, likely due to long-term residency and younger parent age | *Relatively more Turkish parents had a negative perception of the dentist. “Feelings of guilt” and “lack of information”*  *met with the most approval among Turkish parents.*  *More Turkish than German parents had problems making an appointment with a dentist.*  *Low level of comprehension difficulties among the Turkish respondents. German parents also had difficulties in comprehension which might be linked to different use of language rather than language barriers.*  *Very few parents knew about possible co-payments for dental treatment.* |
|  |  |  |  |  | Attitude towards dentists &  Access to Dentist  Knowledge about coverage by health insurance |  |  |

| **Study Type** | **Language** | **Population** | **Group 1** | **Group 2** | **Endpoints** | **Results** | **Conclusions by the authors** |
| --- | --- | --- | --- | --- | --- | --- | --- |
| **Van Steenkiste et al. 2004b**  **Cross-Sectional Epidemiological Study**  **PMID:**  15562346 | German | N=8.096  **Inclusion Criteria:**  Students of first and fourth grade in primary and special needs schools in Rems-Murr-Kreis, Germany  **Age:**  6-7 years or 9-10 years  **Time frame:**  September - July 2000/2001  **Follow-up:**  none | Migrants  N= NA*  (Migrant children: Migrant parents and language spoken at home) | Non-Migrants  N= NA  (German children: German parents (even if only one parent is German)) | Primary:  DMFT and dmft (Mean Values)  Secondary:  age and ethnic distribution  extent of restoration in % calculated as (f MT + f FT]/  f DMFT) × 10.  Percentage of caries free teeth in primary and permanent teeth (age and nationality)  Percentage of Amalgam fillings from all fillings (age and nationality) | - Migrant children had higher caries prevalence and lower restoration rates compared to German peers. | *Higher prevalence of caries and a lower level of remediation among migrants. Migrants*  *do not form a homogeneous group in terms of dental health.*  *The fact that the children of migrants have less restoration and fewer fissure sealings shows that curative or preventive measures that are exclusively are carried out exclusively in the dental practice are used less by migrants.* |
|  |  |  |  |  |  | - Late resettler: 2.8% - Turkish: 6.0% - Ex-Yugoslavia: 1.8% - Italian: 2.4% - Greek: 2.7% - Other: 4.2% - Preventive measures like fissure sealants were less frequently used in migrant children. - children with =/> 1 Fissure Sealing (FS) and average number of Fissure Sealing - 6/7-year-olds (n=4094) - 9/10-year-olds (n=4002) - Caries-free dentition was significantly lower among migrant groups, particularly Turkish and ex-Yugoslavian children. |  |

* NA meaning non-applicable as there was no data available

| **Study Type** | **Language** | **Population** | **Group 1** | **Group 2** | **Endpoints** | **Results** | **Conclusions by the authors** |
| --- | --- | --- | --- | --- | --- | --- | --- |
| **Zeeb et al. 2004**  **Cross-Sectional Survey**  **PMID:**  14994205 | German | N=565  **Inclusion Criteria:**  Adults in Bielefeld, Germany accompanying children to pre-school medical exams. Speaking/writing/reading either German, Polish, Russian or Turkish.  **Age:**  average age: 35.6 years  **Time frame:**  2002  **Follow-up:**  none | Migrants  N= 276  (integrated themselves or parents integrated to Germany) | Non-Migrants  N= 289  (German) | Origin  Social Index (education, employment, size of household/ vocational qualification): missing, low, middle, high  Utilization of dental services over last 6 months  Participation in preventive dental check-ups | Migrants:   - 32.7% Turkey - 22.5% Russia and GUS-countries - 13.6% Est-Europe - 9% South-Europe - 9% Asia - 3.6% other | *The study highlighted the need for improved health communication and culturally tailored education strategies to enhance dental service utilization among migrants*. |
|  |  |  |  |  |  | - Migrants reported less frequent use of dental services, often visiting only in case of acute problems. - Language barriers and limited knowledge of the healthcare system were cited as major obstacles to care. - Cultural differences influenced attitudes toward dental treatment and prevention. - Lower oral health literacy was identified among migrant participants. |  |

**Tab. 2: Search Strategy**

| **Nr.** | **PICO** | **Search Terms** |
| --- | --- | --- |
| **1** | P | Migrant Population  Immigrants  Refugees  MeSH:  Migrant  Immigrant  Refugee |
| **2** | I/O | Oral Health  Dental Care  Health Care Utilization  Dental Check-ups  Dental Caries Risk  Periodontal Disease  MeSH:  Oral Health  Dental Care  Access to Health Care  Dental Caries  Periodontal Disease |
| **3** | C | Native Population  German  Non-migrant  MeSH:  Germany |
| **4** |  | #1 AND #2 AND #3 |
